# DNA copy number profiles and systems biology connect chromatin remodeling and DNA repair in high-risk neuroblastoma

**DOI:** 10.1590/1678-4685-GMB-2024-0007

**Published:** 2024-09-02

**Authors:** Thatyanne Gradowski F. da C. do Nascimento, Joice de Faria Poloni, Mateus Eduardo de Oliveira Thomazini, Luciane R. Cavalli, Selene Elifio-Esposito, Bruno César Feltes

**Affiliations:** 1 Pontifícia Universidade Católica do Paraná, Escola de Medicina, Programa de Pós-Graduação em Ciências da Saúde, Curitiba, PR, Brazil.; 2 Universidade Federal do Rio Grande do Sul, Instituto de Biociências, Departamento de Biofísica, Porto Alegre, RS, Brazil.; 3 Instituto de Pesquisa Pelé Pequeno Príncipe, Curitiba, PR, Brazil.; 4 Georgetown University, Lombardi Comprehensive Cancer Center, Washington, DC, 20007, USA.

**Keywords:** Pediatric cancer, chemotherapy, biological networks, drug resistance, DNA repair.

## Abstract

Neuroblastoma (NB) is a solid tumor that accounts for 15% of all pediatric oncological deaths, and much is due to the low response to therapy in relapsed tumors. High-risk NB may present deletions in chromosome 11q, which may be associated with other chromosomal alterations and a poor response to therapy, but this association is still poorly understood. Using a systems biology network approach, we studied three patients with high-risk NB with deleted 11q stage 4 to highlight the connections between treatment resistance and copy number alterations in distinct cases. We built different protein-protein interaction networks for each patient based on protein-coding genes mapped at the cytobands pre- and post-chemotherapy from distinct copy number alterations data. In the post-chemotherapy networks, we identified five common regulatory nodes corresponding to the gained region located in ch17q:*BIRC5, BRCA1, PRKCA, SUMO2*, and*GPS1*. A crosslink between DNA damage and chromatin remodeling proteins was also found - a connection still poorly understood in NB. We identified a potential connection between *XPB* gain and chemoresistance of NB. The findings help elucidate the molecular profiles of high-risk NB with 11q deletion in pre- and post-chemotherapy tumor samples, which may reflect unique profiles in poor response to treatment.

Pediatric cancer is a rare disease still poorly understood regarding its genetic origin, resistance to treatment, and long-term treatment-associated health complications (Kattner et al., 2019). The heterogeneity of pediatric tumors associated with the difficulty of recruiting a large number of patients for traditional clinical trials shows the importance of precision medicine based on molecular data for more effective treatments and early diagnosis (Westhoff et al., 2018). Neuroblastoma (NB) is a neuroendocrine embryonal tumor originating from neural crest cells during the development of the sympathetic nervous system (Ciaccio et al., 2021). It is the most common extracranial solid tumor diagnosed in early childhood (0-5 years), accounting for 15% of the reported pediatric cancer deaths (Tolbert and Matthay, 2018). Like most pediatric tumors, NB presents predominantly copy number alterations (CNAs), with a low mutation occurrence in primary tumors (Esposito et al., 2017; Mlakar et al., 2017). Among high-risk NB, deletions on the long arm of chromosome 11 (11q deletions) can be found in one-third of the cases associated with tumor recurrence and metastasis development, suggesting similar prognostic significance to *MYCN* amplification (Campbell et al., 2017; Costa and Seuánez, 2018). One of the 11q deleted NBs’ main features is chromosomal instability. However, the 11q region is rarely lost on both chromosomes 11, not fitting the classical single tumor suppressor gene mechanism.

Treatment of high-risk NB usually consists of multiple cycles of induction chemotherapy using platinum drugs, anthracyclines, and alkylating agents (Pinto et al., 2015; Ciaccio et al., 2021) that target signaling pathways, such as DNA repair, cell cycle, and apoptosis. Most patients initially respond to treatment but might become resistant to therapy. Therefore, the integration of patients’ clinicopathological data, molecular tumor signatures, and corresponding enrichment analysis of cell signaling and biological process is a powerful strategy to identify driver genes and/or molecular alterations of tumor resistance and stratify patients and/or groups of patients that may present distinct responses to treatment (Ballerini et al., 2022). Studying large cohorts sometimes can occlude important data, as it can be difficult to discern from the most frequent molecular changes, whereas studying special cases is an efficient way to personalize therapeutic interventions.

Using an integrated strategy of systems biology strategy, we report CNAs of three distinct cases of NB stage 4 with 11q deletion, with paired pre- and post-chemotherapy (CT) primary tumor samples and identify protein-protein interaction (PPI) networks, signaling pathways, and potential molecular targets associated with these high-risk 11q deleted NB patients. These cases were chosen because of their unique characteristics in the cohort as a late relapse behavior, occurring at least two years after the completion of the first line of treatment, their poor response to treatment, and their more distinct NB CNAs profiles among the studied group. 

Patients diagnosed with NB and treated at the Hospital Pequeno Príncipe (HPP), Curitiba, PR, Brazil, between 2004 and 2014 were selected. The study was approved by the Human Research Ethics Committee of the Pontifícia Universidade Católica do Paraná (Curitiba, PR, Brazil) under registration number 3.573.221/2019. The analysis of the human samples used in the present study followed the regulations of the Declaration of Helsinki. The three cases of this study were selected out of an initial group of 96 patients evaluated for CNAs and included due to the presence of 11q tumor deletion (identified in the tumoral samples before CT treatment). Paired tumor samples of all three cases were collected both at diagnosis (pre-CT) and post-CT. This study was approved by the Institutional ethics committee (approval number: 33.573.221) with anonymous patients’ information. The main clinical features of the three patients are presented in Table 1. A summary of the course of the disease and clinical management for each patient is shown in Figure 1A.

**Figure 1-:**
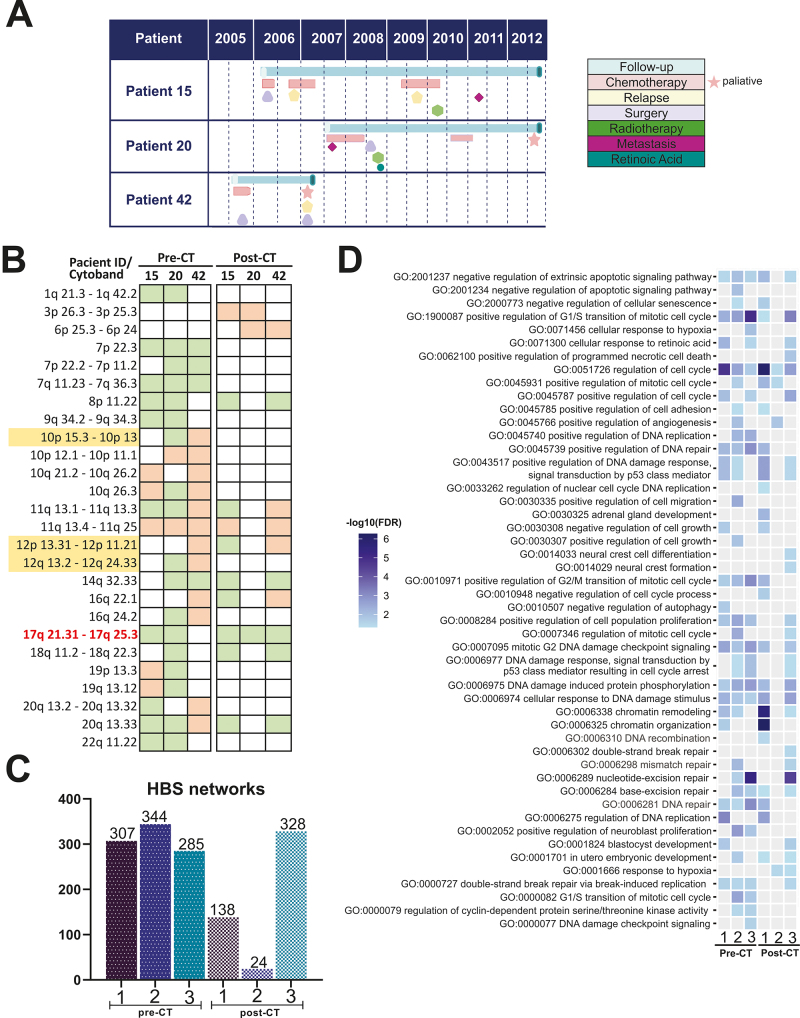
A) Timeline representing the main diagnoses, therapeutic interventions, and outcomes for each patient; B) List of cytobands with copy number alterations (CNA) in the pre-and post-CT tumor samples, affecting at least two patients. Orange and green squares indicate losses and gains, respectively; C) Hub-Bottlenecks-Switches (HBS) in each patient pre- and post-chemotherapy (CT); D) Key gene ontology (GO) terms in pre- and post-CT tumor samples. Only the results that presented a false discovery rate (FDR) < 0.05 were considered. *The regions highlighted in yellow represent regions where the changes were the opposite among the cases analyzed. The regions highlighted in red correspond to where the five identified HBS belong. 17q21.31 (*BRCA1*), 17q25.3 (*BIRC5* and *GPS1*), 17q25.1 (*SUMO2*) and 17p24.2 (*PRKCA*).

**Table 1-:** Summary of the clinical features and outcomes for each case.

Clinical Parameter	Case 1	Case 2	Case3
Age at diagnosis (months)	67	56	6
Shimada histology	Unfavorable	Unfavorable	Unfavorable
MYCN amplification	No	No	No
Primary tumor	Adrenal	Abdominal	Adrenal
Bone marrow infiltration	No	Yes	No
Stage (INSS)	4	4	4
Risk group	High	High	High
Outcome	Dead of disease	Dead of disease	Dead of disease

The patient of case 1 was diagnosed at five years of age. An abdominal tumor was identified with suspected right adrenal NB and bone metastasis. The patient was diagnosed in 02/2006 with nodular ganglioneuroblastoma. Chemotherapy was initiated following the Neuro 2000 protocol for four cycles. Five months later, the disease relapsed. After exploratory laparotomy and suprarenalectomy with complete tumor excision with invasion to the medullary canal, the patient was submitted to the Children’s Cancer Group (CCG) protocol chemotherapy. A second recurrence was observed two years later, despite the chemo and radiotherapy treatment. This patient was followed clinically for six months and died of disease at 14 years old (11/2012).

The patient of case 2 was diagnosed at six years of age. Medullary bone infiltration by undifferentiated neoplasia was detected. This patient was admitted to the hospital for stage 4 NB clinical treatment, presenting iliac bone metastasis and liver and bone marrow infiltration (06/2007). Tumor resection was not conducted due to the adherence of the tumor mass to the renal aorta. The patient initiated the Neuro 2000 protocol CT a month later with two pre-induction and four induction cycles. The patient underwent two surgeries, followed by five cycles of CT. Tumor resection was performed after the second cycle. One year later, consolidation therapy was applied, followed by myeloablative treatment with ten cycles of retinoic acid. Due to the lack of response, the CCG protocol was initiated for four cycles. The patient’s parents did not allow the continuation of treatment with another cycle of CT. Five years later, a relapse occurred. One month later, the patient underwent cardiorespiratory arrest and died at 11 years old (10/2012).

Finally, the patient of case 3 was diagnosed at six months of age with a tumor in the adrenal gland (07/2005). Retroperitoneal tumor resection of the contiguous organ was performed. The patient was diagnosed with normocellular bone marrow with a representation of all three hematopoietic lineages and staggered maturation, with no neoplasia. The Neuro 2000 protocol was initiated two weeks after diagnosis with four complete chemotherapy cycles. Nearly two years later (02/2007), the patient was admitted with stage 4 NB, with an extensive abdominal mass and renal compression. Palliative chemotherapy was administered with admission to the intensive care unit for two days, followed by cardiorespiratory arrest and death (03/2007). 

DNA copy number analysis was performed using the oligonucleotide aCGH platform (SurePrint G3 Human CGH Microarray 8x60K; Agilent Technologies Inc., Santa Clara, CA, USA) according to our previous protocols for formalin-fixed paraffin-embedded samples (Santos et al., 2008; Torresan et al., 2014). DNA was isolated using the standard phenol-chloroform method. Reference DNA was prepared from ten healthy donors’ peripheral blood pools. Equal amounts of tumor and reference genomic DNA (1-2 µg) were digested and enzymatically labeled using the SureTag Complete DNA Labeling Kit (Agilent Technologies, Inc., Santa Clara, CA, USA) and hybridized to the arrays. The array data were analyzed with Feature Extraction v.10.10 software and Agilent CytoGenomics v.3.0, using the ADM-2 algorithm threshold 6.0 and an aberration filter with a minimum of > 3 probes. Copy number alterations (CNA) with gains and losses were defined as minimum average absolute log2 ratio (Cy5 intensity/Cy3 intensity) values of >0.25 and <−0.25, respectively, as per Agilent Cytogenomics guidelines. The number of “calls” (a total significant number of CNAs) and affected cytobands were obtained from the generated aberration interval base reports (Agilent Cytogenomics v. 5.0) (Agilent Technologies, Santa Clara, CA, USA). 

The criteria for the analyzed cytobands were all those present in the three pre-CT tumor samples, including losses and gains. The data was obtained from the Agilent probe report of each case, and any data that were not protein-coding genes were excluded (ex: ORFs, miRNAs). The genes mapped at the selected regions with CNAs of each condition (pre-CT and post-CT) for each patient were used to create a PPI network using the STRINGDB R package (Szklarczyk et al., 2021). The interactions were extracted from the STRING v11.5 database for *Homo sapiens* (Szklarczyk *et al.,* 2019). The following parameters were considered: (i) connections based on co-expression, co-expression-transferred, database, database-transferred, experiments, and experiments-transferred, and (ii) a confidence score filter ≥ 0.4 in each channel. After filtering, the remaining interactions were used as inputs to create an undirected network using the Igraph R package (Csardi and Nepusz, 2006). The igraph ‘simplify’ command removed self-loops and multiple edges. Nodes that were not connected to the main network were removed. For the centrality analysis, the degree, eigenvector, and betweenness measures were calculated. Nodes with above-average degree scores are named “hubs” and indicate a high number of direct interactors, which can be interpreted as fundamental nodes for the network connectivity. Nodes with above-average betweenness scores are called “bottlenecks”. They are known to control the information flow between clusters, thus, being “bridging nodes” that connect one or more bioprocesses between themselves. They can be interpreted as messenger nodes, transporters, or signaling molecules. Finally, nodes with above-average eigenvector scores were named “switches.” This score considers the number of neighbors, the betweenness score, and the same scores of the neighboring nodes. Hence, these nodes can be interpreted as “regulators of regulators.” Nodes with above-average scores for all three metrics were named “hubs-bottlenecks-switches” (HBS) (Scardoni and Lau, 2012).

GO enrichment analyses were performed using the R topGO package (Alexa and Rahnenfuhrer, 2022) using Fisher’s exact test. Only the results with a false discovery rate < 0.05 were considered. Generic GO that does not convey meaningful biological information, such as “regulation of biological processes” and “biochemical processes,” were excluded. We preferred a more precise GO that indicated how a process was regulated, such as “positive/negative regulation of the cell cycle,” when available. GOs annotations, *per se*, are heterogeneous (i.e., based on who, how, and the conditions in which they were annotated); we listed multiple similar incidences to provide a more robust understanding of the bioprocesses within the network.

Array-CGH analysis was performed in DNA from tumor samples obtained at diagnosis (pre-CT) and after induction therapy (post-CT). Cytobands with CNAs in pre- and post-CT were listed (Figure 1 B), and PPI networks were built for each sample. As expected, CNAs were distinct in pre- and post-CT.

The final networks comprised thousands of genes, resulting in ~2.500 up to 4.300 proteins. To narrow down disease-driver genes in NB of interest, we applied a centrality analysis to determine nodes with higher topological relevance by considering three metrics (degree, betweenness, and eigenvector). In PPI networks, nodes with higher topological relevance are usually regulators of biological processes (Scardoni and Lau, 2012), and comparing the topology of disease networks is an efficient and recognized way to uncover potential molecular mechanisms and disease-driven genes (Barabási et al., 2011; Vidal et al., 2011; Menche et al., 2015). Using networks to elucidate molecular changes based on chromosome aberrations was also previously employed (Corrêa et al., 2019, 2021). Hence, due to the cell heterogeneity and selective pressure exerted by CT, cells post-CT, together with their unique molecular profile, remain as the predominant cell type after treatment (as opposed to the ones that were predominant pre-CT), which enforces the necessity to identify new disease-driver regulators to elucidate the molecular mechanisms behind cell survival and maintenance.

All pre-CT networks displayed higher nodes and interconnectivity than the paired post-CT networks. The tumor samples from Case 1 presented 307 HBS pre-CT and 138 HBS post-CT; from Case 2, 344 HBS pre-CT and 24 HBS post-CT were identified; and from Case 3, 285 HBS pre-CT and 328 HBS post-CT (Figure 1 C  and Figure 2). Thus, the same tendency observed for the networks was reflected in the centrality analysis, where larger and more robust networks had higher quantity HBS, and less connected networks displayed fewer HBS. Cases 1 and 2 presented 105 common HBS pre-CT and seven post-CT, while Cases 1 and 3 presented 68 common HBS pre-CT and 39 post-CT (Figure 1 C ). Tumor samples from cases 2 and 3 presented 89 common HBS pre-CT and only one post-CT common HBS (all Ch17q gain). Thus, considering post-CT HBSs only, tumors from Cases 1 and 3 presented more CNA in common when compared to tumors from Cases 2 and 3 or Cases 1 and 2. It can be noted that all patients displayed less HBS in common post-CT, suggesting that the changes in molecular profiles post-CT can lead to unique patterns of major regulators of molecular pathways. This can be especially true due to the use of different chemotherapeutic drugs.

**Figure 2-:**
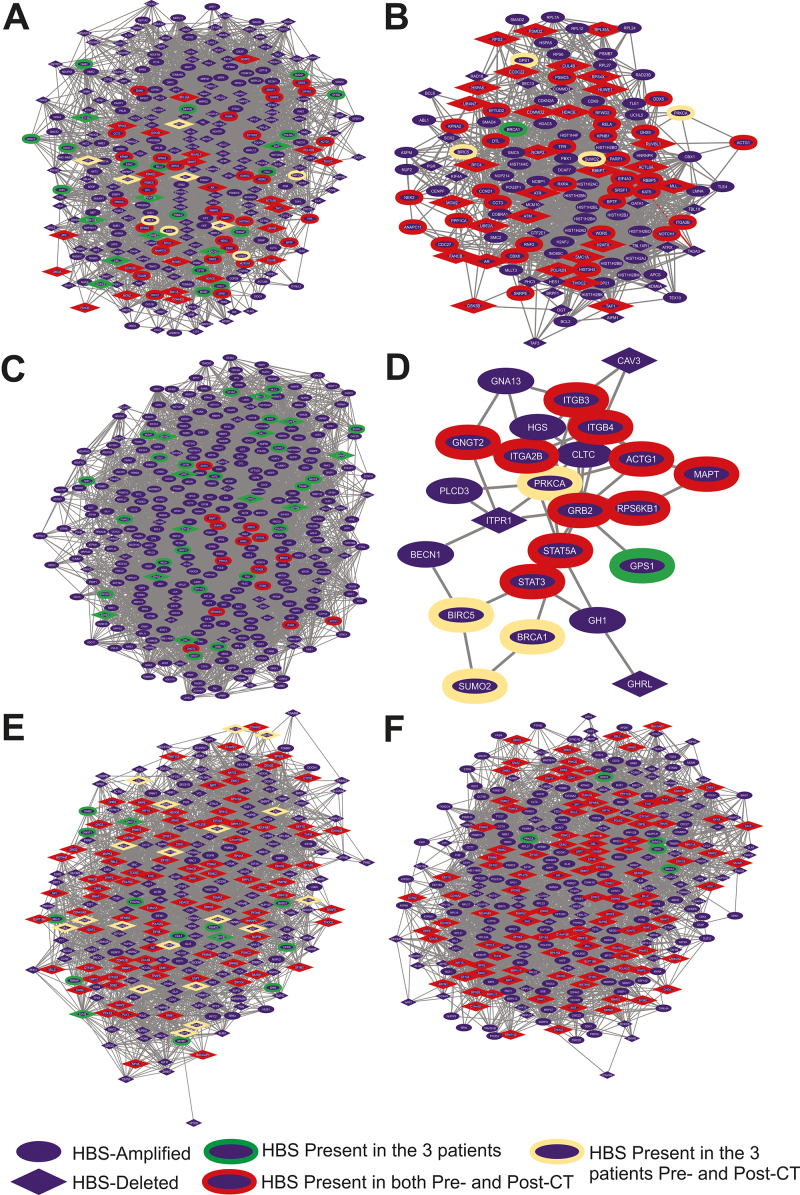
HBS subnetworks in each patient. A, B) Case 1’s HBS subnetwork pre- (307 genes) and post-CT (138 genes), respectively; C, D) Case 2’s HBS subnetwork pre- (345 genes) and post-CT (24 genes), respectively; E, F) Case 3’s HBS subnetwork pre- (285 genes) and post-CT (328 genes), respectively.

In total, 33 HBS were common among the three patients’ tumors pre-CT and five post-CT (*BIRC5, BRCA1, PRKCA, SUMO2,*and*GPS1*) (Figure 2)*.* No HBS was shared among all the patients’ tumor samples when comparing both pre- and post-CT analyses. These results suggested that the overall molecular interactions and the potential disease-driver genes are expected to be unique before and after CT. This agrees with our study - cases with distinct characteristics will not simultaneously overlap their molecular signatures pre- and post-CT. Likewise, it agrees with the variety of treatment outcomes observed in clinical practices, where patients with apparently similar clinical features might not respond to the same treatment in the same fashion. Because our focus was to understand disease-driver genes that might be related to poor response to treatment and disease relapse, we focused on the HBS in common post-CT for further discussion.

In agreement with the previous results, GOs for all patients were also overall heterogenic (Figure 1 D). Most processes are, in some way, related to cancer biology or NB. For example, we found multiple processes associated with proliferation, chromatin remodeling, p53 signaling, and protein phosphorylation (Figure 1 D). However, some bioprocesses highlighted the severity of NB. For example, except for Case 2 post-CT, all cases consistently showed GO associated with a negative regulation of apoptosis pre- and post-CT. However, Case 2 was the only one presenting a positive angiogenesis regulation post-CT, consistent with cancer development and aggressiveness (Viallard and Larrivée, 2017). Case 2 showed the least connected network after CT, with the most drastic change in topology; hence the lack of more detailed GOs is expected. Nevertheless, this also indicates the importance of observing the global shift in molecular interactions in clinical studies, as it can provide significant hints regarding the response to treatment. At last, the obtained GOs agreed with the poor response to the treatment of the studied patients.

Remarkably, Case 3 showed a high enrichment significance for the nucleotide excision repair pathway (NER) (Figure 1 D). Among the HBS of this GO, *XPB* was the only one found in the gained chromosomal region pre- and post-CT. Furthermore, while all samples exhibited significant enrichment for the double-strand break (DSB) repair pathway pre-CT, Case 3 was the only one in which the involvement of this pathway was maintained post-CT. Case 3 was also the only one that presented the pathways of mismatch repair, neural crest formation, neural crest differentiation, and blastocyst development post-CT. Other processes that caught our attention were chromatin remodeling, which was heavily enriched only in Case 1 post-CT (Figure 1 D), and response to hypoxia, which was only present in Cases 2 and 3 post-CT. Thus, we combined these more unique GOs with the five HBS in common between all cases post-CT for further discussion.

Loss of heterozygosity in 11q is one of the main events associated with poor prognosis in NB (Mlakar et al., 2017). In high-risk cases, this deletion is anti-correlated with *MYCN* amplification, an independent prognostic factor in NB, being almost mutually exclusive (Campbell et al., 2017). Here, we presented three cases of stage 4 NB with an 11q deletion, non-amplified *MYCN*, with pre-and post-CT tumor CNA data, where all patients displayed a poor response to treatment and CT resistance. Five HBS were commonly observed, all located in ch17q. Finally, we found a connection between chromatin remodeling and DNA repair, which may be linked to resistance to treatment in these specific cases. Most GOs were expected in the context of cancer and NB. However, the NER process is still poorly understood in NB.

One determining factor of NB is the location of the primary tumor, which differs among patients, and tumors involving the adrenal glands’ medulla are associated with a worse prognosis (Vo et al., 2014). Age at diagnosis, however, is one of the most determining factors of NB aggressiveness, with patients older than 18 months having a higher risk of death (Pinto et al., 2015; Matthay et al., 2016). This particularity is reflected in Case 2 overall survival, where all available treatments still led to tumor recurrence. By studying these unique cases, it is possible to understand molecular variations that would be occluded in larger cohorts. It is fairly reasonable to assume that the lack of chromosomal regions leads to defects in the molecular machinery due to the absence of the genes within the regions. For amplifications, it is also reasonable to assume that such aberrations lead to some level of overexpression of the genes within the region, either directly or indirectly, as it was previously described for the *MYC* oncogene, even though it can not be said that all genes located in the amplified region would be overexpressed (Wang et al., 2006). In this sense, CNAs are known to affect gene expression in NB in different ways (Wang *et al.,* 2006; Fischer et al., 2010; Mlakar et al., 2017).

In Case 1, the total number of CNAs increased post-CT (from 20 to 27). Some GOs, such as cell cycle alterations, were expected within the biological context of tumor progression. However, others, such as adrenal gland development, chromatin remodeling, and DNA repair found in the post-CT tumor sample (Figure 1D), were highly enriched for this patient. Case 2, in contrast, showed a CNA number decrease in the post-CT tumor sample (from 45 to 7) and a poorly connected PPI network (Figure 1 B). This patient was the only one who underwent pre-induction CT with topotecan, a topoisomerase inhibitor, and cyclophosphamide, an alkylating agent that may be related to this patient’s unique molecular profile. Interestingly, proteins codified by genes located in the deleted region in 11q pre-CT, such as *CHEK1, H2AFX*, and *MRE11A*, are targeted by chemotherapeutics, such as cyclophosphamide, in pathways that trigger DNA repair (Voelcker, 2020). Increasingly new functions and relationships have been discussed for DNA repair and chromatin remodeling in the context of NB (Hu et al., 2019), indicating that in our cases, non-amplified MYCN may be affecting these pathways.

In Case 3, major changes in the CNA profile were found, as demonstrated by the increase in the number of CNAs in the post-CT tumor sample (from 18 to 28). Also, in this case, the deletion of 1p36 and 4p16 and gain of 2p24 occurred post-CT (Figure 1 B). This profile change may contribute to increased tumor aggressiveness, considering that genes at the 1p36 region, such as*ARID1A,* are associated with chromatin remodeling. Chromatin-modifying proteins such as ARID1A (1p36 amplified in pre-CT in Case 2) and ARID1B are strongly related to the increased rate of cell proliferation in the NB sympathoadrenal lineage (Shi et al., 2020). Notably, Case 3 had the shortest overall survival (2 years), not completing the entire cycle of CT treatment (induction and consolidation), and was the only one that showed a high enrichment significance for the NER pathway (Figure 1 D). In this sense, major proteins of this pathway, such as DDB2, DDB1, CETN2, XPB, and XPF, appear as HBS for this patient (Figure 2 E ,F). More importantly, DDB2, DDB1, XPF, and CETN2 were deleted in the tumor samples pre-and post-CT, while XPB, a helicase of the TFIIH complex, was amplified in both samples. Although XPF polymorphisms are associated with an increased risk of NB (Zhuo et al., 2018) and the NER pathway is related to chemoresistance to cisplatin (Duan et al., 2020), there is no data in the literature associating XPB and NB. Interestingly, overexpression of XPD, another subunit of the TFIIH complex, is associated with cisplatin resistance (Aloyz et al., 2002). In this sense, overexpressed XPD also stimulated the homologous repair-DSB pathway (Aloyz *et al.,* 2002), which appeared in Case 3 post-CT GO (Figure 1 D). Thus, XPB is a potential target for further studies on cisplatin chemoresistance in patients with NB.

In NB primary tumors, alterations in 17q are frequently identified (approximately 50%) (Uryu et al., 2017). In the present work, the common post-CT HBS found among the three patients are located in ch17q (gain) (e.g.,*BIRC5, BRCA1, PRKCA, SUMO2,*and*GPS1*) (Figure 2). No work in the literature connects the genes presented in this work from the chromosome 17q region and chromatin remodeling and DNA repair in the context of CT response in NB. *BIRC5*encodes the survivin protein, which promotes escape from cell death (Costa and Seuánez, 2018) and may be associated with increased angiogenesis and resistance to treatment (Frazzi, 2021). Furthermore, the activation of BRCA1 leads to the activation of H2AFX (Takaoka and Miki, 2018). However, H2AFX was not present in the studied samples as it is codified in the 11q deleted region, thus, not activating this particular DNA damage response. BRCA1 has been presented as a possible biomarker for survival after cisplatin-based chemotherapy for non-small cell lung cancer (Taron et al., 2004) but not in NB. PRKCA is a serine/threonine kinase involved in tumor development and progression, playing a role in cell survival, proliferation, differentiation, and adhesion (Singh et al., 2017). In lung adenocarcinoma, its high expression was an unfavorable characteristic leading to tumor progression (Jiang et al., 2019). SUMO2, from the SUMO pathway, when upregulated, has been associated with metastasis development and a worse prognosis (Kukkula et al., 2021). Finally, *GPS1* encodes the constitutive photomorphogenesis protein nine signalosomes (CNS1), a multicomplex of eight units implicated in cell cycle control and transcriptional activation (Lee et al., 2011; Fu *et al.,* 2020). It was associated with hepatocellular carcinoma, with inhibition associated with the prevention of progression and metastasis (Fu *et al.,* 2020). Furthermore, a hypoxic microenvironment, a GO term observed in Cases 2 and 3 pre-CT (Figure 1 D), with low pH and nutrient deficiency, may exacerbate this characteristic (Li et al., 2021). Advanced tumors often display proteins involved in the DNA damage response, suggesting that inactivation of this pathway is a prerequisite for tumor progression (Guan et al., 2021; Li *et al.,* 2021). 

NB is a rare tumor; thus, obtaining samples is difficult, especially with paired pre- and post-chemotherapy. Although we explored the molecular profile that might be associated with resistance to chemotherapy in unique patients from our cohort, there are limitations regarding the complete picture of such a profile. In this sense, even though amplifications and deletions are more frequent than mutations in NB, we did not access the mutational profile of these patients. Therefore, we lack data on the full spectrum of genetic aberrations in these patients that could aid in understating their resistance to chemotherapy. Future perspectives of our study include analyzing the gene expression of the targets found in our network analysis, especially *XPB*, and whole genomic sequencing of these patients.

The identified PPI networks and corresponding HBS involved in DNA damage and chromatin remodeling may contribute to the patients’ distinct responses to treatment. However, due to the heterogeneity of deletions and amplifications pre- and post-CT, the diversity of affected bioprocesses might reflect CNAs not necessarily associated with 11q deletion. Multiple deletions and amplifications are shared between two patients pre- and post-CT, which could also play a role in the GO observed in our work. Our results suggested that molecular interactions and disease-driver genes are expected to be unique before and after CT. It agrees with the variety of treatment outcomes, where patients with apparently similar clinical features might not respond to the same treatment in the same fashion. Therefore, the characterization of these molecular profiles is of clinical relevance, considering that it can contribute to tailoring treatment regimens and predict treatment response and recurrence accordingly. Likewise, we argue that integrative systems biology approaches could be favorably associated with clinical and molecular data to enrich the discussion of individual studies, especially those associated with rare diseases, where patients’ data is scarce. 
